# Performance Evolution and Balance in the Curing Mechanism of Inorganic Thermal Insulation Mortar: A Review

**DOI:** 10.3390/ma19143068

**Published:** 2026-07-16

**Authors:** Miaorui Fu, Pinghua Zhu, Feifei Jiang, Jialei Wang, Ronggui Liu, Jiangpei Zhu

**Affiliations:** 1 School of Civil Engineering, Nantong Institute of Technology, Nantong 226002, China; 2 School of Urban Construction, Changzhou University, Changzhou 213164, China

**Keywords:** thermal insulation mortar, fire performance, interfacial transition zone, geopolymerization, hydration reaction

## Abstract

Inorganic thermal-insulation mortars can effectively reduce the energy consumption and carbon emissions of both existing and new buildings while maintaining the thermal stability of building envelopes. Compared with conventional mortars, these materials exhibit more pronounced multiscale coupling during curing, and their microstructural evolution and macroscopic properties are highly sensitive to environmental variables, particularly temperature, humidity, and ionic concentration. This review systematically summarizes the effects of high-temperature curing, high-humidity curing, artificially introduced ions, and special curing regimes on the mechanical properties, durability, thermal conductivity, and fire resistance of inorganic thermal-insulation mortars. The reviewed studies indicate that hydration, geopolymerization, and CO_2_-curing reactions can all promote microstructural densification and thus enhance mechanical performance and durability. Elevated temperature and humidity generally accelerate reaction kinetics, intensify internal hydration, and facilitate the generation and deposition of gel products, thereby refining the pore structure and improving strength development. However, the same densification process may also increase the continuity of the solid phase and form more effective heat-transfer pathways, which is unfavorable for thermal-insulation performance. Mildly alkaline curing environments can further stimulate binder reactions and improve matrix compactness, although excessive ionic activity may negatively affect pore stability and long-term performance. Among the coupled curing conditions, wet–dry cycling appears to provide a more favorable balance between mechanical-property development and pore-structure preservation, because periodic humidity gradients can enhance strength formation, stabilize the interfacial transition zone, and reduce cracking sensitivity. Overall, the effect of curing on inorganic thermal-insulation mortars is governed by the competition and balance between reaction enhancement, pore-structure evolution, and interfacial stabilization. Future curing design should therefore focus on system-dependent optimization to achieve a rational balance among mechanical performance, thermal insulation, and fire resistance.

## 1. Introduction

Building energy consumption and carbon emissions have become critical issues in the pursuit of sustainable development in the construction sector [1,2,3]. Because the thermal performance of building envelopes directly affects indoor thermal comfort and building energy demand under different climatic conditions, improving the insulation capacity of building materials has become an important strategy for reducing operational energy consumption in both existing and new buildings [4,5]. In this context, inorganic thermal-insulation mortars have attracted increasing attention owing to their combined advantages in durability, flame resistance, and thermal stability [6]. As energy-efficient envelope materials, they have been widely applied in building retrofitting and other engineering practices requiring improved thermal performance [7,8,9].

Inorganic thermal-insulation mortars are commonly prepared by incorporating lightweight aggregates such as expanded vermiculite, expanded perlite, vitrified microbeads, and silica aerogel into cementitious matrices [10,11]. Owing to the porous nature and distinct physicochemical characteristics of these aggregates, such mortars can achieve low density and favorable thermal-insulation performance [12,13]. However, these same features also introduced intrinsic limitations. In practical applications, the loose and highly voided structure of lightweight aggregates often weakened their interfacial bonding with the cement-based matrix, increased water absorption, and reduced mechanical strength. Although aggregate modification could partially improve interfacial performance [14], curing regulation provided an economical route for improving the overall performance of inorganic thermal-insulation mortars.

**Figure 1:**
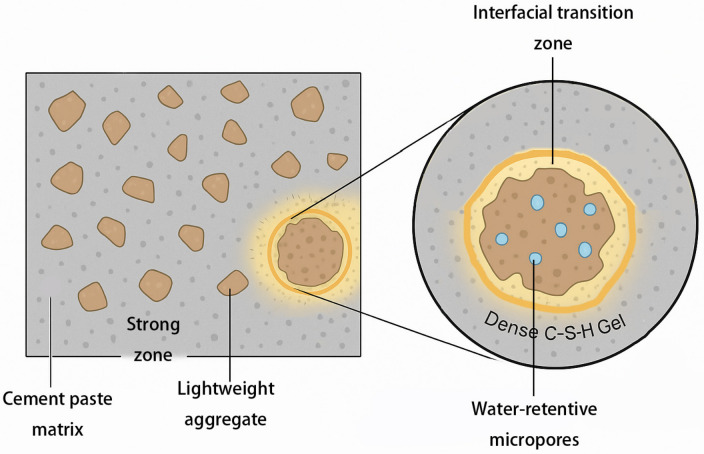
Schematic diagram of inorganic insulation mortar structure.

Curing plays a decisive role in the microstructural development and property evolution of inorganic thermal-insulation mortars. During curing, environmental variables such as temperature, humidity, curing duration, and ionic concentration directly affect hydration and geopolymerization reactions, the formation of gel products, pore-structure evolution, and the stability of the interfacial transition zone (ITZ) as shown in Figure 1 [15,16,17,18]. As a consequence, curing conditions strongly influence not only mechanical strength and durability, but also thermal conductivity and fire resistance [19,20]. However, existing studies have mainly focused on mix design, aggregate characteristics, and modification techniques, whereas the curing mechanism of inorganic thermal-insulation mortars has not yet been systematically reviewed. More importantly, the effects of curing cannot be interpreted simply as uniformly beneficial, because reaction enhancement and structural densification may improve mechanical properties while simultaneously compromising thermal-insulation performance. In view of this, the review summarizes the effects of high-temperature curing, high-humidity curing, artificially introduced ions, and special curing regimes on the mechanical properties, thermal-insulation performance, and fire resistance of inorganic thermal-insulation mortars, with emphasis on the underlying microstructural mechanisms and the performance trade-offs involved. The aim is to provide a clearer theoretical basis for the curing-oriented design and engineering application of such materials.

## 2. Effect of Curing Mechanisms on Mechanical Properties

### 2.1. Effect of High-Temperature Curing

**Figure 2:**
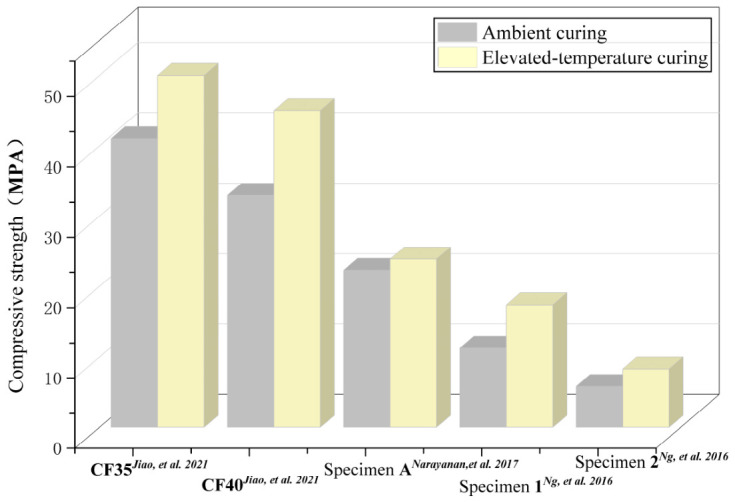
Effect of Curing Temperature on the Compressive Strength of Specimens [23,24,25].

High-temperature curing exerts a significant influence on the mechanical properties and durability of inorganic insulating mortars. In general, increasing the curing temperature accelerates hydration and geopolymerization, promotes the early formation of reaction products, and enhances the early-age strength of the material. However, this beneficial effect is not unlimited. Once the curing temperature exceeds the optimum thermal window of a given material system, long-term strength retention, pore-structure stability, and durability may deteriorate. Therefore, the effect of high-temperature curing on insulating mortars should be understood as a temperature-dependent balance between reaction enhancement and microstructural instability, as shown in Figure 2 [21,22].

Compared with continuous standard curing, steam preconditioning generally produces a more pronounced increase in early-age strength. For example, fly ash–slag geopolymer mortars exposed to steam curing at 60 °C for 24 h and then subjected to standard curing exhibited compressive strengths 2.2–5.1 times higher than those of specimens cured only under standard conditions [23]. In addition, after 300 freeze–thaw cycles, these thermally treated specimens maintained a relative dynamic elastic modulus above 85%, whereas the standard-cured specimens exhibited a loss of approximately 60%, indicating that appropriate high-temperature curing can improve not only strength development but also structural integrity and durability.

A similar trend has been reported for recycled powder (RP) and fly ash (FA)-containing systems. In expanded perlite insulating mortars activated by NaOH, curing at 90 °C for different durations promoted the early formation of a more continuous and stable gel network, which in turn created favorable conditions for the sustained development of later-age mechanical properties. Within a curing period of 90 d, prolonging the thermal-curing duration increased the flexural strength by up to 173% [26]. Likewise, in FA-based geopolymer insulating mortars subjected to four different curing regimes, specimens cured in a hot-air oven exhibited the highest 28 d compressive strength, representing a 31% increase over naturally cured specimens [24]. These results indicate that elevated-temperature curing can effectively accelerate geopolymerization, densify the internal structure, and improve the mechanical performance of geopolymer-based insulating mortars.

For aerogel-containing plaster (ACP), elevated temperature also generally promotes strength development. Comparative studies of different curing methods have shown that the compressive strength of most ACP specimens increased by more than 25% under high-temperature curing conditions. Nevertheless, the beneficial effect was not monotonic: as the treatment temperature increased from room temperature to 105 °C, compressive strength generally increased, whereas a further increase to 120 °C led to a decline [25]. This finding again suggests that high-temperature curing is beneficial only within an appropriate temperature range and that excessive thermal exposure may impair the stability of the developing microstructure.

Despite the clear advantages of thermal activation, several studies have also reported adverse effects of excessive high-temperature curing. In expanded shale insulating mortars, higher curing temperatures improved early-age strength but also accelerated moisture evaporation [27], increased porosity, and promoted the formation of thermally induced microcracks [28]. Temperature gradients generated during steam curing may induce internal thermal stresses, thereby weakening later-age strength and durability. Similarly, prolonged exposure to high-temperature curing may intensify the drying and water loss of hydration products and geopolymeric gels, reduce dry density, and ultimately lead to a more pronounced decline in the mechanical properties of inorganic insulating mortars [29,30]. These observations indicate that the positive effect of thermal curing on reaction kinetics may be offset by pore coarsening, moisture loss, and microstructural damage when the curing temperature or duration becomes excessive.

Overall, the mechanical response to high-temperature curing is strongly material-dependent. For geopolymer-based insulating mortars containing FA, RP, or expanded perlite, moderate elevated-temperature curing is generally beneficial because it accelerates geopolymerization and promotes early structural build-up. By contrast, for systems prone to moisture loss and thermal cracking, such as expanded shale mortars, excessive temperature or prolonged heating may coarsen the pore structure and weaken long-term mechanical stability. Therefore, the role of thermal curing should be interpreted in relation to material type and optimum thermal window rather than as a uniformly beneficial factor.

### 2.2. Effect of High-Humidity Curing

High-humidity curing is another key factor governing the mechanical performance of inorganic insulating mortars. Sufficient humidity ensures the continuous progression of hydration reactions and provides a favorable environment for the migration, deposition, and filling of hydration products within the pore network [31,32,33]. Compared with natural curing, high-humidity curing generally improves both compressive and flexural strength [34], because the cementitious matrix can more effectively penetrate the pore structure of lightweight aggregates such as RP and vitrified microbeads, thereby strengthening ITZ bonding and enhancing the overall compactness and durability of the material [35].

For inorganic insulating mortars, the beneficial effect of humidity is not limited to conventional external curing. Owing to their porous structure, lightweight aggregates can absorb water during curing and release it gradually, thereby acting as internal curing reservoirs. Although such porosity generally reduces strength, it also improves the adaptability of insulating mortars to high-humidity environments. Under these conditions, resistance to water-vapor transport is reduced, hydration proceeds more continuously, and the matrix becomes denser and more compact. In RP insulating mortars, sufficiently reacted hydration products can fill internal pores after curing, thereby compensating for part of the mechanical loss and improving durability [36]. Similarly, in expanded shale insulating mortars, the high water-retention capacity of the lightweight aggregate provides additional internal moisture, while the dense ITZ formed by hydration products further supports the enhancement of mechanical strength and durability [28]. Humidity also contributes to shrinkage mitigation and microstructural stabilization. A high-humidity internal environment can alleviate shrinkage and cracking induced by moisture loss, increase the degree of hydration, reduce the proportion of open porosity, and promote the formation of a denser microstructure. As a result, not only mechanical strength but also resistance to chloride ion penetration can be improved [25]. A similar positive effect has also been reported for ACP. After being stored at 20 °C for 24 h, aerogel-based specimens cured for 28 d in water at 80 °C exhibited higher compressive and flexural strengths than those cured in a dry environment at the same temperature. This improvement was mainly attributed to the more complete hydration of the cement-based matrix and the formation of a dense hydration-product layer under humid conditions [37].

However, the effect of high-humidity curing is not uniformly positive, and several studies have reported contrasting results. In RP specimens directly exposed to a hot–rain cycling environment after 24 h of storage at room temperature, pore volume first decreased and then increased with increasing cycle number [38]. In the early stage, normal hydration reduced porosity, whereas in the later stage, fragmentation of RP particles increased the proportion of large pores and interconnected pore networks, resulting in strength reduction. Likewise, in aerogel–silica fume insulating mortars, wet–dry cyclic curing produced higher compressive strength than continuous water curing when the aerogel content was relatively low, whereas prolonged high-humidity curing produced adverse effects when the aerogel content was high [39,40]. These results suggest that the effect of humidity depends on the balance between hydration enhancement and the preservation of a stable pore structure.

The favorable role of wet–dry cyclic curing is particularly noteworthy. In aerogel–silica fume systems, silica fume can fill part of the pore structure and reduce permeability under cyclic humidity variation. Repeated drying and rewetting promote internal moisture evaporation and reabsorption, which in turn stimulates hydration, reduces capillary and macropores, and enhances both compressive and flexural properties [41,42]. Therefore, for some insulating mortars, especially systems with aerogel or other highly interface-sensitive lightweight aggregates, wet–dry cyclic curing may provide a more favorable balance between strength development and pore-structure integrity than continuous high-humidity curing.

**Figure 3:**
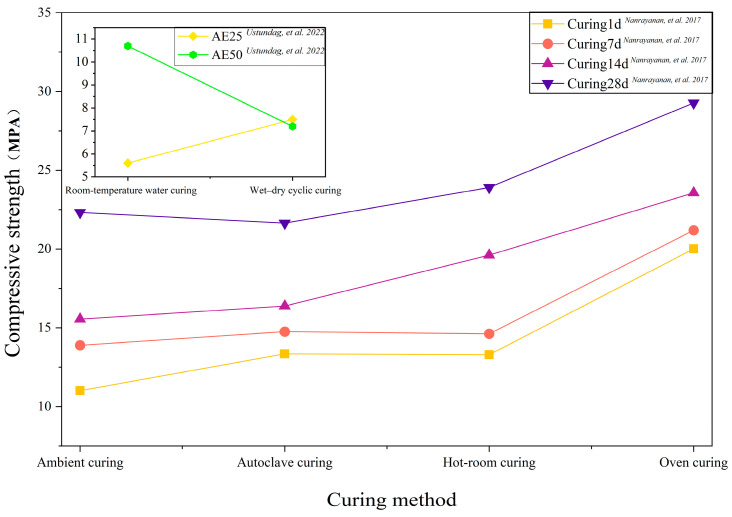
Effect of Curing Humidity on the Compressive Strength of Specimens [24,39].

**Figure 4:**
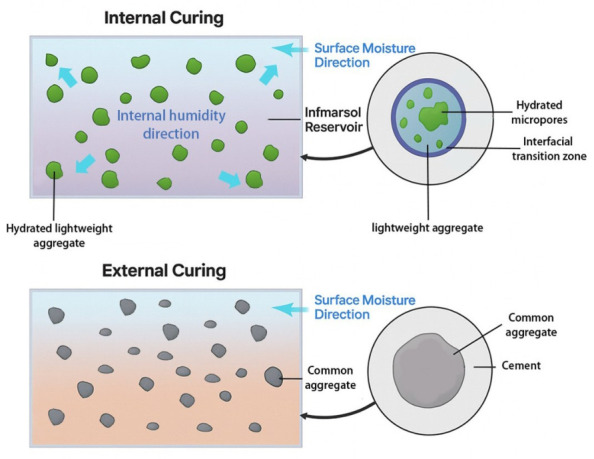
Schematic diagram of the difference between ordinary curing and internal curing.

Overall, current studies indicate that the influence of humidity on insulating mortars is governed mainly by its effect on hydration continuity, internal curing, and pore filling. In contrast to conventional mortars, inorganic insulating mortars exhibit a more distinctive internal-curing behavior because lightweight aggregates can absorb moisture and delay its release, thereby maintaining internal humidity and promoting continuous hydration and geopolymerization. This mechanism explains why humidity control is particularly critical for such materials. At the same time, wet–dry cyclic curing can mitigate drying-induced microcracking, preserve microstructural integrity, and improve long-term mechanical performance. Therefore, from the perspective of mechanical-property optimization, humidity regulation should not be understood simply as maintaining a continuously saturated environment; instead, a system-dependent curing strategy that balances reaction enhancement and pore-structure stability is more appropriate for inorganic insulating mortars, as illustrated in Figure 3 and Figure 4.

### 2.3. Effect of Artificially Introduced Ions in the Curing Environment

Inorganic insulating mortars are characterized by a loose and porous microstructure, which makes their early-age densification and interfacial development particularly sensitive to the ionic environment during curing [43,44]. Deliberately introducing alkaline ions (e.g., Ca^2+^ and Li^+^) into the curing environment can promote early-stage hydration and gel formation, thereby accelerating densification and enhancing structural stability. However, this approach is also associated with an evident upper bound: excessive introduction of highly active ions, especially Na^+^, may increase internal microcracks and voids, reduce compactness, and ultimately weaken long-term mechanical performance. Therefore, ion-assisted curing should be regarded as a balance between reaction activation and microstructural integrity rather than a uniformly positive strategy.

For RP geopolymer insulating mortars, immersion in dilute HCl or CaCl_2_ solution for 6 h has been reported to improve both mechanical properties and durability. This improvement is mainly attributed to ion-exchange and stabilization effects: Na^+^ in the pore structure is replaced by H^+^ or Ca^2+^, leading to the formation of products such as NaCl and CaCO_3_, which suppress subsequent reactions of Na^+^ with CO_2_ and water that would otherwise generate Na_2_CO_3_·H_2_O and damage the gel framework. As a result, the deterioration of RP geopolymer insulating mortars under hygrothermal conditions can be mitigated and strength can be improved [45].

In aerogel–silica fume systems, cyclic curing in a 13% MgSO_4_ solution (one week) followed by oven curing at 105 °C (one week), repeated for eight cycles, resulted in a 24.6% increase in compressive strength. Under MgSO_4_ curing, continuous exposure to Mg^2+^ promoted the formation of insoluble salts, reduced pore-water content, and increased structural compactness. In addition, Mg^2+^ reacted with cement hydration products to form more stable structures, which contributed to improved resistance to chemical attack and enhanced compressive strength [39].

Another approach is to introduce alkaline activators directly into the curing environment. Rashad [46], following methodologies used for low-thermal-conductivity concretes [47,48,49], introduced NaOH and other alkaline activators into the curing environment of RP insulating mortars to activate slag-, silica fume-, and FA-containing constituents. H_2_O_2_ was used as a foaming agent during preparation to maintain the required porosity. After curing and subsequent accelerated aging, the results showed that the mechanical properties and durability were partially improved, indicating that external activator ions can enhance reaction extent and microstructural optimization in porous insulating mortars.

Overall, available studies suggest that a reasonable increase in alkaline-ion concentration during curing can optimize microstructure, reduce crack risk, and improve the overall performance of insulating mortars. This ion-assisted curing strategy is considered economical and practical, particularly for applications requiring enhanced durability. Nevertheless, because excessive ionic activity may trigger microcracking and pore instability, the selection of ion type, concentration, and exposure mode should be regulated to ensure that reaction activation does not come at the expense of long-term compactness and mechanical stability.

### 2.4. Effects of Special Curing Mechanisms

Conventional steam curing at elevated temperature can accelerate early reaction and strength development; however, prolonged exposure may generate relatively wide microcracks at the cement–aggregate interface and reduce durability. In this context, alternative curing technologies have been explored to achieve rapid hardening while mitigating thermal-gradient-related damage.

Direct electric curing (DEC) [50,51] accelerates hardening by applying electric voltage to cementitious materials, inducing collisions among charged ions and generating Joule heat. Compared with conventional curing, DEC is associated with a lower environmental burden and has been considered more environmentally friendly [52]. In one reported procedure, specimens were treated under alternating current (50 Hz, 30 V) for 6 h, followed by cooling in a closed insulated chamber for 24 h. Relative to standard curing, DEC markedly enhanced early-age compressive strength and accelerated solidification; for some specimens, the 1 d compressive strength increased by as much as 65.74%. Meanwhile, the specimens exhibited a denser internal structure, and the uniform, gradual temperature rise effectively controlled expansion, reduced microcrack formation, and improved durability [27].

CO_2_ pretreatment represents another special curing route. Desulfurized gypsum provides alkaline metal ions (e.g., Ca and Mg) that can react with CO_2_ to form carbonates, thereby increasing dry density and enhancing mechanical properties. For ACP, three groups of specimens were subjected to CO_2_ pretreatment for 0, 3, and 7 d, followed by standard curing to 28 d. The introduced CO_2_ filled micropores in aggregates and accelerated the overall hydration of ordinary Portland cement and recycled concrete powder. During this process, Ca(OH)_2_ and C_2_S were converted into CaCO_3_, which improved surface appearance, reduced defects such as honeycombing and pitting, and increased compressive strength by 11.3% [53].

In addition to DEC and CO_2_ pretreatment, other special curing methods, such as microwave curing [47,54] and vacuum curing [48], have been proposed for improving mechanical performance. However, their applicability to insulating mortars remains insufficiently validated, and further systematic investigations are required to clarify their effectiveness, practicality, and performance limits in the context of porous lightweight-aggregate systems.

## 3. Effect of Curing Mechanisms on Thermal Insulation Performance

### 3.1. Effect of High-Temperature Curing

**Figure 5:**
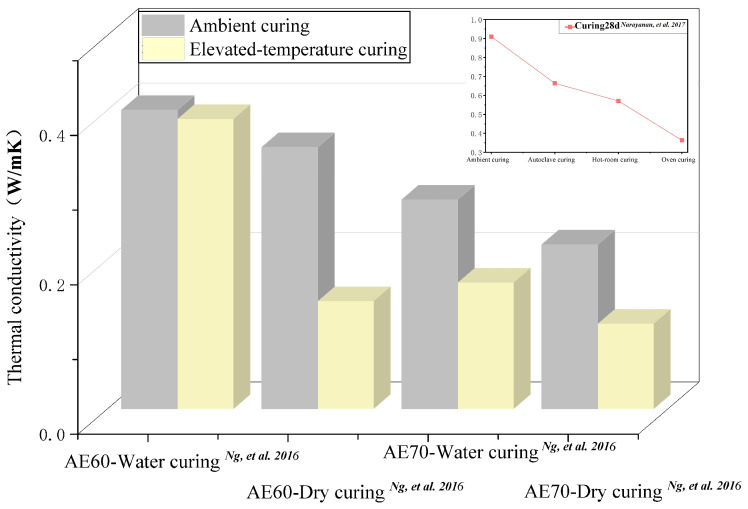
Effect of Curing Temperature on the Thermal Conductivity of Specimens [24,25].

Specimens cured under elevated-temperature conditions generally exhibit lower thermal conductivity, indicating an improvement in thermal insulation performance, as shown in Figure 5. For specimens containing 70% aerogel by volume, the minimum thermal conductivity was obtained under dry curing at 120 °C [25]. A similar tendency was reported for geopolymer systems: among the tested curing regimes, FA-based geopolymer mortar cured in a hot-air oven showed the best thermal insulation performance, with a thermal conductivity of 0.363 W/m·K, which was significantly lower than those obtained under the other curing conditions [24]. These results suggest that high-temperature curing can modify the internal structure of inorganic insulating mortars in a manner favorable to reducing heat transfer.

For vitrified microbead geopolymer insulating mortars, storage at 60 °C for 24 h prior to standard curing was also reported to be beneficial for reducing thermal conductivity [49]. At this elevated-temperature stage, geopolymerization was effectively promoted, resulting in a reduction in overall porosity and the formation of a more continuous solid-phase structure. Such structural evolution is conducive to compressive-strength development and can suppress heat transfer associated with air movement inside the pore system [55]. In addition, the porous structure of vitrified microbeads can hinder crack propagation at elevated temperatures, thereby contributing to the overall thermal stability of the material. However, the effect of elevated-temperature curing is not unidirectionally beneficial. Although high temperatures can accelerate hydration and geopolymerization reactions in insulating mortars, the resulting strength enhancement is often accompanied by reduced porosity and a possible increase in thermal conductivity [56].

### 3.2. Effect of High-Humidity Curing

**Figure 6:**
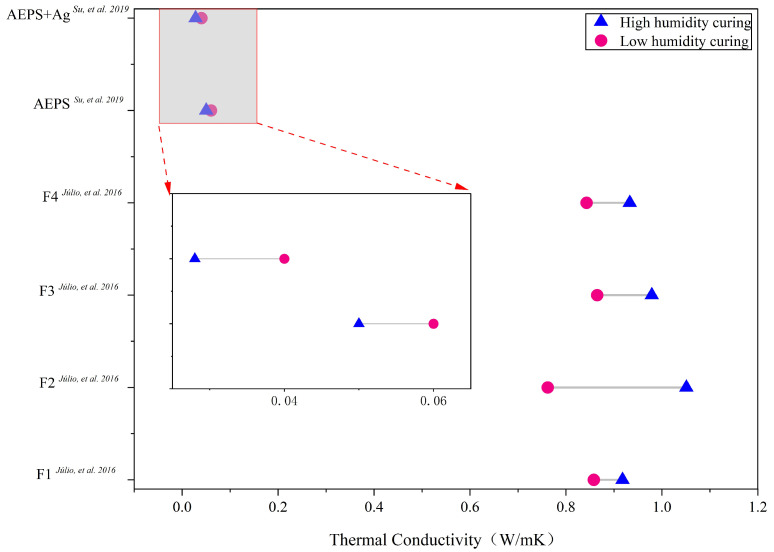
Effect of Curing Humidity on the Thermal Conductivity of Specimens [49,60].

As shown in Figure 6, high-humidity curing generally exerts a positive effect on the thermal insulation performance of inorganic insulating mortars by influencing pore preservation, moisture migration, and the distribution of hydration products. During curing, a humid environment can prevent the premature closure of the pore structure of lightweight aggregates, thereby maintaining relatively high porosity, reducing thermal conductivity, and improving thermal insulation performance. For example, when waste rubber powder is used as a fine aggregate, its incorporation forms elastic centers that help preserve a favorable pore structure during moisture evaporation. Under high-humidity conditions, this contributes to the formation of intact and uniformly distributed micropores within the mortar matrix while avoiding the extensive development of harmful defects such as interconnected pores and densely distributed cracks [57].

A similar trend has been observed in other modified insulating mortars. When a small amount of calcined clay is used to replace part of the cement clinker, hydration products can fill some of the pores during curing; however, because the hydration reaction does not proceed completely, the porosity and water-absorption capacity remain at relatively high levels [58]. In EPS/vitrified microbead insulating mortars, thermal insulation performance and mechanical properties can be balanced through curing in water at room temperature. In such systems, emulsified asphalt may be used as a polymer modifier, and with the addition of 0.2% air-entraining agent, the thermal conductivity decreases by 30% to 0.065 W/(m·K) [59]. These findings indicate that the positive role of humidity is closely associated with its interaction with supplementary materials and admixtures, which together regulate the pore network and heat-transfer pathway.

For silica fume-based insulating mortars, specimens stored at room temperature for two days and then subjected to standard curing until 28 d also showed the beneficial role of a humid environment [61]. Maintaining the specimens at a relative humidity above 95% effectively prevented moisture loss, while silica fume promoted secondary hydration reactions. The resulting products were mainly flocculent or plate-like and exhibited a porous structure, thereby lowering the overall heat-transfer efficiency [62]. This result further supports that high-humidity curing can improve thermal insulation performance when the curing environment promotes the formation of a stable and finely distributed pore system rather than excessive matrix densification.

However, the effect of high-humidity curing should be interpreted separately for different material categories rather than generalized for all insulating mortars. For conventional porous lightweight-aggregate systems, such as EPS/vitrified microbead, silica-fume-modified mortars, and some expanded perlite-based systems, a humid curing environment is generally beneficial because it sustains hydration, promotes gradual pore filling, and helps maintain a relatively stable and fine pore network [63,64]. In these systems, the improvement in thermal insulation is mainly associated with pore preservation and the formation of a more uniform microstructure rather than with simple matrix densification.

In contrast, aerogel-based systems exhibit a distinctly different response to humidity. To ensure the thermal stability of ACP, ACP specimens containing EPS were first moist-cured for 7 d and then naturally cured for 21 d, which resulted in relatively low thermal conductivity [65]. Júlio [60] cured ACP at 20 ± 2 °C and a relative humidity of 65 ± 5% to ensure sufficient moisture participation in the reaction process. Zaidi [66] further showed that, for aerogel–fly ash insulating mortars, specimens stored at 25 °C and 50% relative humidity for 7 d and then naturally cured exhibited thermal conductivity values of 0.762–0.865 W/(m·K), while specimens water-cured at room temperature for 7 d and then naturally cured for 21 d showed values of 0.75–0.973 W/(m·K). By contrast, continuous water curing for 28 d increased the thermal conductivity to 0.933–1.051 W/(m·K). These results indicate that prolonged moist curing is unfavorable for maintaining the thermal insulation performance of aerogel-containing systems.

This difference is closely related to aggregate–matrix compatibility. Aerogel is highly sensitive to humidity because of its hydrophobic nature and highly porous structure. Under prolonged moist curing, the inorganic aerogel skeleton may separate from the surrounding cement paste, resulting in a wider ITZ than that observed in conventional lightweight-aggregate mortars. Such interfacial defects are much less pronounced in expanded perlite-based systems. Therefore, the role of humidity should be classified according to material type: continuous humid curing is generally suitable for conventional porous lightweight-aggregate mortars, whereas staged curing or wet–dry cyclic curing is more appropriate for aerogel-based mortars, in which preservation of interfacial stability is more critical than maximizing hydration.

### 3.3. Effect of Artificially Introduced Ions in the Curing Environment

Artificially introduced alkaline ions can significantly influence the thermal insulation performance of inorganic insulating mortars by regulating hydration kinetics, pore-structure evolution, and the type and distribution of hydration products. Because these ions can readily come into contact with the reactants in the curing environment and act as external activators, they promote the hydration of slag-containing constituents and increase the formation of products such as C–S–H gel. When 0.03% Li_2_CO_3_ was used as an activator, the mortar reached a 28 d compressive strength of 34.1 MPa, while maintaining a thermal conductivity of 1.32 W/m·K [19]. This result indicates that an appropriate dosage of lithium carbonate can improve mechanical performance without compromising favorable thermal properties.

A similar effect was observed under CaCl_2_ treatment. After 28 d of curing, the thermal conductivity decreased, while the compressive strength increased by 25.71%, indicating simultaneous improvement in thermal stability and mechanical performance [45]. This treatment inhibited the penetration of Na^+^ into the gel network through the formation of compounds such as CaClOH and CaCO_3_. Meanwhile, these reaction products also filled part of the pore structure, thereby significantly enhancing specimen stability and thermal insulation performance. These findings suggest that appropriate ionic intervention can beneficially modify both the chemical environment and the microstructure of porous insulating mortars.

Under the curing conditions reported by Ustundag [39], the lowest thermal conductivity, 1.458 W/m·K, was obtained for specimens containing 0.25% aerogel under MgSO_4_ curing. In a high-humidity environment, hydration products can migrate and fill part of the pore system of the mortar, thereby reducing pore volume and affecting the formation of larger pores and air voids. As a result, the median pore diameter decreases and the total porosity is altered, which exerts a beneficial effect on thermal conductivity. To avoid the alkaline reaction between silica aerogel and cement, aerogel is generally incorporated together with silica fume, or special aerogel-based composites are prepared [67]. During curing and service, silica fume acts as a sacrificial filler to preserve both the mechanical performance and the thermal insulation capacity of the mortar [68].

Overall, ions introduced into the curing environment affect the thermal insulation performance of mortar mainly by modifying the hydration rate, the pore structure, and the distribution of solid reaction products. In mortars containing lightweight aggregates, a relatively high proportion of fine pores is present within the pore system. Under alkaline ionic curing, the pore structure can be modified, particularly within the pore-diameter range of 4–8 nm, where hydroxides and other solid products may form inside nanoscale pores and thereby reduce effective heat-transfer pathways. Fine and uniformly distributed pores are beneficial for suppressing heat-flow transmission and enhancing the thermal insulation performance of mortar. Therefore, by rationally selecting the type and concentration of ions, it is possible to regulate the microscopic pore structure of mortar and simultaneously improve mechanical performance and thermal insulation properties. In addition, the combined use of aerogel and silica fume can effectively avoid performance loss caused by alkaline reactions, thereby maintaining the thermal insulation capacity of the mortar while improving its mechanical properties.

### 3.4. Effects of Special Curing Mechanisms

Among special curing methods, Direct electric curing (DEC) is generally unfavorable for the retention of thermal insulation performance. Compared with steam-cured specimens, specimens subjected to DEC exhibit lower porosity [27]. Because DEC refines the pore structure and reduces the overall porosity of the specimens, it may decrease the number of pores that are beneficial for suppressing heat transfer, thereby adversely affecting the thermal insulation performance of mortar.

For CO_2_ curing, mortar porosity generally decreases with increasing curing duration. In insulating mortars in which recycled concrete powder partially replaced cement, the thermal conductivity of specimens subjected to 7 d of CO_2_ curing increased from 0.051 W/m·K to 0.057 W/m·K [53]. This increase was mainly attributed to the formation of CaCO_3_, which filled the pores in the mortar and thereby increased thermal conductivity. However, some studies have suggested a potentially beneficial effect of carbonation curing on thermal conductivity. Under CO_2_ gas pressure, part of the air inside the specimen may be displaced and replaced by CO_2_; because the thermal conductivity of CO_2_ at room temperature is 0.014 W/m·K, which is lower than that of air (0.024 W/m·K), the overall thermal conductivity of carbonated specimens may decrease [62]. These findings indicate that the effect of CO_2_ curing on thermal insulation performance remains dependent on the competition between pore filling and gas-phase replacement.

In summary, conventional high-temperature steam curing is prone to coarsening the pore structure and generating significant temperature gradients inside the specimens, which may lead to inconsistent expansion of different hydration products and consequently produce more interconnected pores and large pores. By contrast, direct electric curing and carbonation curing can effectively avoid such temperature-gradient-related effects, but they may also increase the thermal conductivity of the specimens and thereby impair thermal insulation performance. Therefore, the application of special curing methods in inorganic insulating mortars should be evaluated not only in terms of reaction acceleration and strength development, but also from the perspective of pore-structure control and heat-transfer regulation.

## 4. Effect of Curing Mechanisms on Fire Resistance

### 4.1. Effect of High-Temperature Curing

In studies on inorganic insulating mortars, thermal insulation performance can, to some extent, reflect fire resistance. Correspondingly, curing temperature exerts an important influence on the fire resistance of these materials, mainly by affecting the reactions occurring during curing as well as the compactness, strength, porosity, and thermal stability of the matrix [69]. In general, the role of elevated-temperature curing in fire resistance should be understood as a balance between reaction enhancement and microstructural stability under subsequent high-temperature exposure.

The beneficial effect of elevated-temperature curing has been demonstrated in several insulating mortar systems. For FA-based geopolymer insulating mortars, fire resistance was evaluated using an electric resistance furnace, and the residual compressive strength was measured after cooling. Among the different curing regimes, specimens cured in a hot-air oven exhibited the highest residual compressive strength, indicating superior resistance to fire exposure [24]. A similar trend was observed in expanded vermiculite insulating mortars. Compared with air-cured specimens, thermally cured specimens showed lower mass loss and higher residual strength after high-temperature exposure. Even at 750 °C, although a certain degree of strength loss occurred, the thermally cured specimens still maintained relatively favorable fire resistance [70]. Chindaprasirt [71] also used a curing regime involving oven curing at 60 °C for 24 h followed by storage at 25 °C and 50% relative humidity, where the relatively high curing temperature was adopted to ensure the fire resistance of specimens containing waste automotive glass as lightweight aggregate.

However, the positive effect of high-temperature curing is not unlimited. For expanded perlite insulating mortars subjected to preliminary treatment at 90 °C for different durations, specimens thermally treated for 24 h exhibited greater flexural-strength loss after exposure to 400 °C and 600 °C than those cured for 4 h and 8 h, indicating that excessively prolonged thermal curing may adversely affect high-temperature resistance [72]. This suggests that although elevated-temperature curing can promote early structural build-up, excessive thermal exposure during curing may impair the later fire resistance of the material.

The underlying mechanism is closely related to the dual effect of thermal activation on reaction development and pore-structure evolution. Under elevated-temperature conditions, the reactivity of certain solid-waste-based materials can be enhanced. For example, alumina and silica in waste brick powder are more likely to react under thermal activation, generating more gel products and rapidly completing structural build-up within the first few hours. Nevertheless, when high-temperature pretreatment becomes excessive, the resulting increase in porosity may exceed the pore-filling capacity of geopolymer gels, thereby inducing pore enlargement, structural loosening, and microcrack formation. Such microstructural degradation readily reduces both flexural and compressive strength and ultimately weakens fire resistance.

Overall, the influence of high-temperature curing on fire resistance is strongly system-dependent. Insulating mortars containing different lightweight aggregates, such as expanded vermiculite, waste brick powder, and waste automotive glass, differ in thermal response, water-absorption behavior, and interfacial characteristics, and therefore exhibit different optimal curing temperatures and curing durations. As the duration of thermal curing increases, substantial moisture evaporates from the mortar, which increases pore content and microstructural heterogeneity. Nevertheless, elevated-temperature curing can effectively shorten the curing time required for geopolymer materials under ambient conditions and thus provides practical industrial advantages, including reduced reaction time, higher production efficiency, and lower curing-related cost and time demand [73]. Therefore, from the perspective of fire-resistance-oriented design, the key issue is not whether high-temperature curing should be used, but how to control its intensity and duration within a system-appropriate range.

### 4.2. Effect of High-Humidity Curing

High-humidity curing affects fire resistance mainly through its influence on ITZ development, hydration-product formation, pore evolution, and high-temperature phase stability. In this context, the compatibility between the cementitious matrix and lightweight aggregates is particularly important. When incompatibility exists, as in systems containing paraffin and magnesium hydroxide, the resulting voids and the phase transition of the aggregates at elevated temperatures can help maintain the residual compressive strength of the mortar and thereby enhance fire-resistant performance. In addition, a significant synergistic effect exists between humidity and material composition. For example, under high-humidity conditions, ettringite formed in calcium aluminate cement can effectively improve the fire resistance of mortar, while some moist-cured mortars may form new minerals such as gehlenite at elevated temperatures, which can fill microcracks and pores, preserve compressive strength, and thus contribute to favorable fire performance.

Experimental evidence further supports the beneficial role of humid curing in some systems. For slag–pumice mortars cured under natural water-curing conditions, the ITZ between the cementitious matrix and aggregates exhibited stronger bonding after exposure to elevated temperatures [74]. Similarly, Yıldırım et al. [75], using the mix proportions and curing regime reported by Turkmen [76], cured specimens at a relative humidity of 100% and a temperature of 23 ± 2 °C before high-temperature testing. The results showed that high-humidity curing promoted the formation of more hydrates such as ettringite, which increased porosity and improved fire resistance. When paraffin-encapsulated zeolite was used as the lightweight aggregate, full cement hydration under a high-humidity curing environment enabled C–S–H and Ca(OH)_2_ to fill part of the pore structure. After 28 d of water curing, Ca(OH)_2_ diffraction peaks were detected in all specimens, confirming that hydration proceeded sufficiently and generated the key reaction products [77].

However, the beneficial effect of high-humidity curing is not universal, especially for aerogel-containing systems. For ACP, a longer moist-curing duration leads to the formation of more hydration products, which refine the pore structure and render the paste denser. Tunnel fire simulation tests, in which a curing regime involving alternating moist curing and dry curing under natural conditions was compared with standard curing, showed that the denser microstructure produced under moist-curing conditions improved the mechanical properties of ACP but also increased its thermal conductivity, thereby reducing its fire resistance. Under high temperatures, ACP subjected to moist curing developed longer and wider microcracks, which accelerated damage to the cement paste. By contrast, specimens subjected to alternating wet–dry curing exhibited the most favorable fire performance.

Overall, the effect of high-humidity curing on fire resistance is governed by the balance between hydration enhancement, interfacial stabilization, pore-system evolution, and thermal transport behavior at elevated temperatures. For many insulating mortars, humid curing promotes sufficient hydration, improves ITZ bonding, and enables the formation of hydration products or high-temperature phases that help preserve residual strength after fire exposure. However, for ACP and other highly interface-sensitive systems, prolonged moist curing may produce an excessively dense microstructure, increase thermal conductivity, and aggravate crack development under fire conditions. Therefore, with respect to fire resistance, humidity regulation should also be system-dependent. In some cases, alternating wet–dry curing may provide a more favorable compromise between mechanical integrity, thermal insulation, and fire performance than continuously humid curing.

## 5. Conclusions

(1) A shorter period of medium–high temperature curing is usually beneficial for geopolymer-based and porous lightweight aggregate mortar, as it accelerates reaction kinetics and promotes the formation of early structures.
(2) Traditional porous lightweight aggregate mortar usually benefits from high-humidity curing, while mortar based on aerogel is more sensitive to long-term moisture exposure, because interface defects may occur between hydrophobic aerogel skeleton and cement matrix, which is more suitable for alternate dry and wet curing or controlling the humidity of the curing environment.
(3) The densification induced by maintenance can damage the thermal insulation performance, depending on whether it reduces the connected pores and moisture-related heat transfer, or creates a continuous solid heat transfer pathway.
(4) A small amount of Ca^2+^, Mg^2+^, LI^+^ in the maintenance environment can promote partial mortar reaction activation and improve matrix density. CO_2_ solidification and direct electro-solidification can improve early strength or reduce damage associated with temperature gradients.

## 6. Perspectives

(1) Future research should further clarify the coupled relationships among reaction processes, microstructural evolution, and performance development. Because the formation of C–S–H gel, geopolymeric products, and related phases is highly complex, their migration, deposition, and pore-filling behavior remain difficult to quantify. More systematic studies are therefore needed to reveal how hydration and geopolymerization govern thermal insulation, mechanical properties, and durability, and to support the establishment of a more predictive theoretical framework for curing-oriented design.
(2) Special curing methods such as direct electric curing and CO_2_ curing require further evaluation. Although these methods have shown potential in pore-structure regulation and strength control, their economic feasibility, fire-resistance performance, and long-term influence on thermal insulation remain insufficiently understood. Future work should combine microstructural characterization with long-term performance assessment to determine their actual applicability and limitations.
(3) Research on other non-conventional curing methods remains limited. Approaches such as microwave curing and vacuum curing may affect internal reaction kinetics and pore evolution through rapid dehydration, selective heating, or pressure regulation, but their effects on thermal insulation, fire resistance, and interfacial stability have not yet been systematically clarified. Further studies should integrate experiments, simulations, and engineering-scale validation to assess their practical potential.
(4) Future curing research should move from single-property evaluation toward integrated design. Instead of focusing only on isolated improvements, curing strategies should simultaneously consider reaction kinetics, pore-structure preservation, ITZ stability, thermal conductivity, and fire resistance, so as to develop system-specific and application-oriented curing regimes and advance the field from empirical optimization to mechanism-guided design.

## Data Availability

No new data were created or analyzed in this study. Data sharing is not applicable to this article.

## References

[1] Huang H., Wang H., Hu Y.-J., Li C., Wang X. (2022). The development trends of existing building energy conservation and emission reduction—A comprehensive review. Energy Rep..

[2] Ahmed S., El Attar M.E., Zouli N., Abutaleb A., Maafa I.M., Ahmed M.M., Yousef A., Ragab A. (2023). Improving the thermal performance and energy efficiency of buildings by incor-porating biomass waste into clay bricks. Materials.

[3] Li Y., Wang J., Lin X., Wang H., Li H., Li J. (2022). Purification effects of recycled aggregates from construction waste as constructed wetland filler. J. Water Process Eng..

[4] Li X., Zhou Y., Yu S., Jia G., Li H., Li W. (2019). Urban heat island impacts on building energy consumption: A review of approaches and findings. Energy.

[5] Zhao H., Magoulès F. (2012). A review on the prediction of building energy consumption. Renew. Sustain. Energy Rev..

[6] Cabeza L.F., De Gracia A., Pisello A.L. (2018). Integration of renewable technologies in historical and heritage buildings: A review. Energy Build..

[7] Zhang X., Nie S., He M., Wang J. (2021). Energy-saving renovation of old urban buildings: A case study of beijing. Case Stud. Therm. Eng..

[8] Reza-E-Rabbi S., Bhuiyan M.A., Zhang G., Dodampegama S., Atapattu K. (2026). Simulation-and Metamodel-Based Multi-Objective Optimization for Sustainable Building Retrofit Across Climatic Conditions. Materials.

[9] Miao Y., Ye T., Xiao J., Lau S.S.Y., Zhou Z. (2024). Investigation on alkali-activated insulation mortar containing high-volume recycled concrete powder for energy-efficient buildings. Energy Build..

[10] Chen H., Shen G.Q., Feng Z., Liu Y. (2024). Optimization of energy-saving retrofit solutions for existing buildings: A multidimensional data fusion approach. Renew. Sustain. Energy Rev..

[11] Huo H., Deng X., Wei Y., Liu Z., Liu M., Tang L. (2024). Optimization of energy-saving renovation technology for existing buildings in a hot summer and cold winter area. J. Build. Eng..

[12] Abu-Jdayil B., Mourad A.-H., Hittini W., Hassan M., Hameedi S. (2019). Traditional, state-of-the-art and renewable thermal building insulation materials: An overview. Constr. Build. Mater..

[13] Posani M., Veiga R., de Freitas V.P. (2021). Retrofitting historic walls: Feasibility of thermal insulation and suitability of thermal mortars. Heritage.

[14] Huang J., Wang S., Teng F., Feng W. (2021). Thermal performance optimization of envelope in the energy-saving renovation of existing residential buildings. Energy Build..

[15] Jiang D., Lv S., Cui S., Sun S., Song X., He S., Zhang J., An P. (2020). Effect of thermal insulation components on physical and mechanical properties of plant fibre composite thermal insulation mortar. J. Mater. Res. Technol..

[16] Zhang S., Zhang A., Chen B., Dai H. (2026). Performance and Mesoscopic Simulation of Self-Compacting Concrete Made with Different Lithological Types of Manufactured Sand. Buildings.

[17] Ma G., Yan L., Shen W., Zhu D., Huang L., Kasal B. (2018). Effects of water, alkali solution and temperature ageing on water absorption, morphology and mechanical properties of natural FRP composites: Plant-based jute vs. mineral-based basalt. Compos. Part B.

[18] Sun Z., Mingming W. (2019). Effects of sol-gel modification on the interfacial and mechanical properties of sisal fiber reinforced polypropylene composites. Ind. Crops Prod..

[19] Bostanci L., Sola O.C. (2018). Mechanical properties and thermal conductivity of aerogel-incorporated alkali-activated slag mortars. Adv. Civ. Eng..

[20] Upshaw M., Cai C.S. (2021). Feasibility study of MK-based geopolymer binder for RAC applications: Effects of silica fume and added CaO on compressive strength of mortar samples. Case Stud. Constr. Mater..

[21] Abid M., Zeb S., Shah M.K., Muhammad S., Bashir M.O., Almujibah H., Elshekh A.E.A., Onyelowe K.C. (2025). Enhancing Mechanical Performance and Microstructure of Reactive Powder Concrete Through Optimization of High-temperature Curing Regimes. Case Stud. Constr. Mater..

[22] Yang H., Shen Z., Zhang M., Wang Z., Li J. (2024). Mechanical properties and microstructure of cement-based materials by different high-temperature curing methods: A review. J. Build. Eng..

[23] Jiao Z., Li X., Yu Q. (2021). Effect of curing conditions on freeze-thaw resistance of geopolymer mortars containing various calcium resources. Constr. Build. Mater..

[24] Narayanan A., Shanmugasundaram P. (2017). An experimental investigation on flyash-based geopolymer mortar under different curing regime for thermal analysis. Energy Build..

[25] Ng S., Jelle B.P., Zhen Y., Wallevik Ó.H. (2016). Effect of storage and curing conditions at elevated temperatures on aerogel-incorporated mortar samples based on UHPC recipe. Constr. Build. Mater..

[26] Çelikten S., Erdoğan G. (2022). Effects of perlite/fly ash ratio and the curing conditions on the mechanical and microstructural properties of geopolymers subjected to elevated temperatures. Ceram. Int..

[27] Wang J., Xiang Y., Li Y., Dong R., Xiao Q., Cai Y., Ren X., Long G. (2023). A comparative study on the properties and environmental impact of mortar with the different paste-to-aggregate ratios under direct electrical and steam curing. J. Build. Eng..

[28] Liu C., Yang L., Li Z., Nie S., Hu C., Wang F. (2022). Improve the long-term property of heat-cured mortars blended with fly ash by internal curing. J. Build. Eng..

[29] Zajac M., Hoock S., Stabler C., Haha M.B. (2017). Effect of hydration kinetics on properties of compositionally similar binders. Cem. Concr. Res..

[30] Ge W., Zhang Z., Ashour A., Li W., Jiang H., Hu Y., Shuai H., Sun C., Li S., Liu Y. (2023). Hydration characteristics, hydration products and microstructure of reactive powder concrete. J. Build. Eng..

[31] Hou X., Li J., Xu J., Xiao X., Wang J., Liu Y., Ossman M., Lin F., Wang X., Li Y. (2024). Experimental study of sulfoaluminate cement-based rapid repair mortar undergoing hot/wet harsh conditions: Mechanical strengths, hydration products, and ettringite evolution mechanism. ACS Sustain. Chem. Eng..

[32] Nahata Y., Kholia N., Tank T.G. (2014). Effect of curing methods on efficiency of curing of cement mortar. APCBEE Procedia.

[33] Luo F., Wang J., Zhu C., Yang J. (2026). Research on Early-Age Shrinkage and Prediction Model of Ultra-High-Performance Concrete Based on the BO-XGBoost Algorithm. Materials.

[34] Braiek A., Karkri M., Adili A., Ibos L., Ben Nasrallah S. (2017). Estimation of the thermophysical properties of date palm fibers/gypsum composite for use as insulating materials in building. Energy Build..

[35] Liu S., Li Q., Zhang J., Zhao K., Wang L., Zhang Z. (2024). Research on mechanical properties of high-strength thermal insulation mortar. Constr. Build. Mater..

[36] Vyšvařil M., Pavlíková M., Záleská M., Pivák A., Žižlavský T., Rovnaníková P., Bayer P., Pavlík Z. (2020). Non-hydrophobized perlite renders for repair and thermal insulation purposes: Influence of different binders on their properties and durability. Constr. Build. Mater..

[37] Linnow K., Niermann M., Bonatz D., Posern K., Steiger M. (2014). Experimental studies of the mechanism and kinetics of hydration reactions. Energy Procedia.

[38] Xiong H., Yuan K., Xu J., Wen M. (2021). Pore structure, adsorption, and water absorption of expanded perlite mortar in external thermal insulation composite system during aging. Cem. Concr. Compos..

[39] Ustundag O., Sola O.C., Ustundag O., Sola O.C. (2022). Effect of aerogel/silica fume under curing methods on properties of cement-based mortars. Rev. Constr..

[40] Bostancı L., Ustundag O., Sola O.C., Uysal M. (2019). Effect of various curing methods and addition of silica aerogel on mortar properties. Građevinar.

[41] Li P., Wu H., Liu Y., Yang J., Fang Z., Lin B. (2019). Preparation and optimization of ultra-light and thermal insulative aerogel foam concrete. Constr. Build. Mater..

[42] Al Zaidi I.K., Demirel B., Atis C.D., Akkurt F. (2020). Investigation of mechanical and thermal properties of nano SiO_2_/hydrophobic silica aerogel co-doped concrete with thermal insulation properties. Struct. Concr..

[43] Ayati B., Ferrándiz-Mas V., Newport D., Cheeseman C. (2018). Use of clay in the manufacture of lightweight aggregate. Constr. Build. Mater..

[44] Hu Z., Xiong Y., Cai Y., Zheng S., Lv Y., Li Y., Zhao X., Wang Y., Ma L. (2026). Effect of a Composite Activator on Comprehensive Performance of Alkali-Activated Foam Concrete. Materials.

[45] Gao H., Liao L., Liang Y., Tang X., Liu H., Mei L., Lv G., Wang L. (2021). Improvement of durability of porous perlite geopolymer-based thermal insulation material under hot and humid environment. Constr. Build. Mater..

[46] Rashad A.M., Mosleh Y.A., Mokhtar M.M. (2024). Thermal insulation and durability of alkali-activated lightweight slag mortar modified with silica fume and fly ash. Constr. Build. Mater..

[47] Zhou F., Pan G., Meng H., Mi R. (2022). Effect of secondary curing on the performance of microwave cured concrete. Constr. Build. Mater..

[48] Chang H., Wang X., Wang Y., Li S., Wang J., Liu J., Feng P. (2022). Influence of low vacuum condition on mechanical performance and microstructure of hardened cement paste at early age. Constr. Build. Mater..

[49] Su Z., Guo L., Zhang Z., Duan P. (2019). Influence of different fibers on properties of thermal insulation composites based on geopolymer blended with glazed hollow bead. Constr. Build. Mater..

[50] Ma C., Peng J., Zhou H., Zhou R., Ren W., Du Y. (2021). An effective method for preparing high early-strength cement-based materials: The effects of direct electric curing on portland cement. J. Build. Eng..

[51] Wang J., An J., Li Y., Xiang Y., Xiao Q., Tang Z., Long G. (2024). Influencing factors of the temperature rise of direct electric curing concrete and its effect on concrete properties. Constr. Build. Mater..

[52] Wang J., Long G., Xiang Y., Dong R., Tang Z., Xiao Q., Yang Z., Ma K. (2022). Influence of rapid curing methods on concrete microstructure and properties: A review. Case Stud. Constr. Mater..

[53] Zhou Z., Xiao J., Ye T., Wang J., Choi D. (2024). Influence of recycled concrete powder and CO_2_ curing on the properties of thermal insulation mortars. Constr. Build. Mater..

[54] Suwan T., Paphawasit B., Fan M., Jitsangiama P., Chindaprasirt P. (2021). Effect of microwave-assisted curing process on strength development and curing duration of cellular lightweight geopolymer mortar. Mater. Manuf. Process..

[55] Longo F., Lassandro P., Moshiri A., Phatak T., Aiello M.A., Krakowiak K.J. (2020). Lightweight geopolymer-based mortars for the structural and energy retrofit of buildings. Energy Build..

[56] Hou Y., Wang Y., Shan S., Zhu W., Luo J. (2026). Mechanical Performance and Microstructures of Vitrified Fly Ash Microbeads in Concrete. J. Build. Eng..

[57] Pan Z., Liu F., Li H., Li X., Wang D., Ling Z., Zhu H., Zhu Y. (2024). Performance evaluation of thermal insulation rubberized mortar modified by fly ash and glass fiber. Buildings.

[58] Siline M., Ghorbel E., Bibi M. (2017). Effect of freeze—Thaw cycles on the physicomechanical properties of a pozzolanic mortar. Constr. Build. Mater..

[59] Dong X., Wang S., Gong C., Lu L. (2014). Effects of aggregate gradation and polymer modifiers on properties of cement-EPS/vitrified microsphere mortar. Constr. Build. Mater..

[60] Júlio M.d.F., Soares A., Ilharco L.M., Flores-Colen I., de Brito J. (2016). Silica-based aerogels as aggregates for cement-based thermal renders. Cem. Concr. Compos..

[61] (2023). Research on fire resistance of silica fume insulation mortar. J. Mater. Res. Technol..

[62] Kiran T., Yadav S.K., N A., Mathews M.E., Andrushia D., Lubloy E., Kodur V. (2022). Performance evaluation of lightweight insulating plaster for enhancing the fire endurance of high strength structural concrete. J. Build. Eng..

[63] Zhang H.-Y., Liu J.-C., Wu B. (2021). Mechanical properties and reaction mechanism of one-part geopolymer mortars. Constr. Build. Mater..

[64] Nurruddin M.F., Sani H., Mohammed B.S., Shaaban I. (2018). Methods of curing geopolymer concrete: A review. Int. J. Adv. Appl. Sci..

[65] Gomes M.G., Flores-Colen I., da Silva F., Pedroso M. (2018). Thermal conductivity measurement of thermal insulating mortars with EPS and silica aerogel by steady-state and transient methods. Constr. Build. Mater..

[66] Zaidi A.K.A.A., Demirel B., Atis C.D. (2019). Effect of different storage methods on thermal and mechanical properties of mortar containing aerogel, fly ash and nano-silica. Constr. Build. Mater..

[67] Jia G., Guo J., Li Z. (2023). Controllable preparation of aerogel/expanded perlite composite and its application in thermal insulation mortar. Constr. Build. Mater..

[68] Gao T., Jelle B.P., Gustavsen A., Jacobsen S. (2014). Aerogel-incorporated concrete: An experimental study. Constr. Build. Mater..

[69] Koksal F., Gencel O., Kaya M. (2015). Combined effect of silica fume and expanded vermiculite on properties of lightweight mortars at ambient and elevated temperatures. Constr. Build. Mater..

[70] Koksal F., Bayraktar O.Y., Bodur B., Benli A., Kaplan G. (2023). Insulating and fire-resistant performance of slag and brick powder based one-part alkali-activated lightweight mortars. Struct. Concr..

[71] Chindaprasirt P., Lao-un J., Zaetang Y., Wongkvanklom A., Phoo-ngernkham T., Wongsa A., Sata V. (2022). Thermal insulating and fire resistance performances of geopolymer mortar containing auto glass waste as fine aggregate. J. Build. Eng..

[72] Cong P., Cheng Y. (2021). Advances in geopolymer materials: A comprehensive review. J. Traffic Transp. Eng. (Engl. Ed.).

[73] Wongsa A., Boonserm K., Waisurasingha C., Sata V., Chindaprasirt P. (2017). Use of municipal solid waste incinerator (MSWI) bottom ash in high calcium fly ash geopolymer matrix. J. Clean. Prod..

[74] Aydın S. (2008). Development of a high-temperature-resistant mortar by using slag and pumice. Fire Saf. J..

[75] Yıldırım S., Baynal K., Fidan O. (2019). Internal curing and temperature effect on lightweight and heat insulated mortar with recycled concrete aggregate. Acta Phys. Pol. A.

[76] Türkmen İ., Gül R., Çelik C. (2008). A taguchi approach for investigation of some physical properties of concrete produced from mineral admixtures. Build. Environ..

[77] Yoo D.H., Jeon I.K., Kim H.G., Lee J.S., Ryou J.-S. (2021). Experimental evaluation of fire resistance performance of cement mortar with PCM/mg(OH)2-based composite fine aggregate. Constr. Build. Mater..

